# Effects of sevoflurane and propofol on the development of pneumonia after esophagectomy: a retrospective cohort study

**DOI:** 10.1186/s12871-017-0458-4

**Published:** 2017-12-04

**Authors:** Guo-Hua Zhang, Wen Wang

**Affiliations:** Department of Anesthesiology, National Cancer Center/Cancer Hospital, Chinese Academy of Medical Sciences and Peking Union Medical College, No. 17 Panjiayuannanli Road, Chaoyang District, Beijing, 100021 China

**Keywords:** Sevoflurane, Propofol, Pneumonia, Esophagectomy

## Abstract

**Background:**

Postoperative pneumonia (PP) is one of the common complications following esophagectomy and associated with poor short- and long-term outcomes. Sevoflurane and propofol, which have inflammatory-modulating effects, are common used general anesthetics. This study aimed to compare the effects of anesthesia with sevoflurane and propofol on the development of PP after esophageal surgery for cancer.

**Methods:**

The electronic medical records of patients who underwent elective esophagectomy between July 2013 and July 2016 were reviewed. We conducted univariate and multivariate logistics analysis and propensity score matching analysis to compare the effect of sevoflurane and propofol on the incidence of PP and to identify the risk factors for PP after esophagectomy.

**Results:**

Overall, the incidence of postoperative pneumonia was 9.5%. There was no significant difference in the rates of PP between sevoflurane group and propofol group either before or after propensity score matching (9.6% vs 8.0%, *P* = 0.606; 7.7% vs 6.4%, *P* = 0.754, respectively). Univariate and multivariate analysis revealed that alcohol use (OR 1.513; 95% CI 1.062–2.156), surgical procedure (Sweet: referent; Ivor-Lewis: OR 1.993; 95% CI 1.190–3.337; Three-incision: OR 1.878; 95% CI 1.296–2.722) and surgeon experience (high-volume: referent; low-volume: OR 1.525; 95% CI 1.090–2.135) were significant risk factors of postoperative pneumonia.

**Conclusions:**

Sevoflurane did not differ from propofol in terms of affecting the risk of PP development after esophagectomy.

## Background

Postoperative pneumonia (PP) is one of the most frequently observed complications following esophagectomy for cancer and has a potentially negative impact on short- and long-term outcomes [1–3]. The incidence of PP following esophagectomy ranges from 8.7 to 38% according to many previous studies [2–5]. In recent years, several studies have also been performed to investigate potential risk factors of postoperative pneumonia. Advanced age, greater American Society of Anesthesiologists (ASA) physical status, chronic obstructive pulmonary disease (COPD), smoking history, alcohol use, prolonged operative time, preoperative chemoradiotherapy, type of operation, and high intraoperative blood loss were found to be predictors for developing postoperative pneumonia [4, 6–8].

The high rate of postoperative pneumonia may result from surgical trauma and one-lung ventilation (OLV) [9]. The former can elicit the release of proinflammatory cytokines while the latter induce pulmonary inflammatory reactions [9, 10]. These reactions play an important role in the development of subsequent systemic inflammatory disorders [11, 12]. Although OLV results in alveolar proinflammatory effects, their extent may depend on administration of anesthetics [13]. Sevoflurane and propofol are commonly used general anesthetics and have been shown to modulate the inflammatory responses in experimental and clinical studies [14–18]. However, there have been few clinical studies to investigate which anesthetic can be more helpful to reduce PP after esophageal surgery for cancer.

Thus, our retrospective study aimed to (1) compare the effects of sevoflurane-based inhalational and propofol-based total intravenous anesthesia on the incidence of PP and (2) to identify risk factors of PP following esophagectomy.

## Methods

This is a retrospective cohort study. Patients who underwent elective esophageal cancer surgery under general anesthesia between July 2013 and July 2016 in our hospital were identified through our electronic medical recording system.

We excluded patients with missing data or those who were not anesthetized with sevoflurane or propofol. All eligible patients were divided into two groups based on anesthetic method: sevoflurane group and propofol group. Anesthesia was induced with midazolam, propofol, opioids, cisatracurium/rocuronium in all patients and maintained with sevoflurane or propofol combined with opioids in each group, respectively. During two-lung ventilation and OLV, tidal volume was set to 6–8 mL/kg with positive end expiratory pressure (PEEP) (5–10 cm H_2_O) if necessary and peak inspiratory pressure kept under 35 cm H_2_O. The fraction of inspired oxygen was adjusted to achieve pulse oxygen saturation > 95% and the respiratory rate set to maintain end-tidal CO_2_ concentrations between 35 to 45 mmHg. Generally, neostigmine 2 mg and atropine 1 mg were injected to reverse the action of muscle relaxants at the end of surgery. Most of the patients were routinely extubated at the end of surgery in the operating theatre and then transferred to the post-anesthesia care unit (PACU). They usually stayed in PACU for at least 30 min and were discharged from PACU after reaching the discharge criteria. Only those who did not meet the extubation standards were transferred to intensive care unit (ICU) for mechanical ventilation. Besides, some patients who underwent reoperation or those who developed severe respiratory dysfunction (PaO_2_<60 mmHg and/or PaCO_2_>55 mmHg) due to various causes in surgical ward after surgery were re-admitted to ICU for ventilator support. Some patients transferred to ICU only received close observation and further treatment without being ventilated. Patient-controlled intravenous analgesia was used in both groups.

All studied variables were obtained by reviewing the electronic medical records and divided into baseline characteristics, intraoperative parameters, and postoperative outcomes. A detailed description of studied variables was listed in Table 1. The primary outcome was the occurrence of postoperative pneumonia which was suspiciously diagnosed based on clinical features (body temperature ≥ 38 °C, leukocyte count >11.0 × 10^9^/L or <4.0 × 10^9^/L and purulent secretions) and confirmed by radiographic findings (infiltrative abnormalities) or tracheal aspirates culture [1, 19]. Once diagnosed as pneumonia, patients were generally treated with antibiotics, physical therapy (taking deep breath, coughing, chest percussion, atomization inhalation, aspiration of sputum), and if necessary, mechanical ventilation according to hospital protocol. We only included patients with definite PP diagnosis which was indicated explicitly in the electronic medical records.

**Table 1:** Comparison of preoperative and intraoperative variables before and after propensity score matching between sevoflurane group and propofol group

Variables	Total	Before Matching			After Matching		
	(*n* = 1659)	Group S (*n* = 1571)	Group P (*n* = 88)	*P*-value	Group S (*n* = 78)	Group P (*n* = 78)	*P*-value
Age (years)	60.7 ± 8.4	60.7 ± 8.4	61.0 ± 8.1	0.740	60.9 ± 8.0	60.7 ± 8.1	0.890
Male	1383 (83.4)	1304 (83.0)	79 (89.8)	0.097	65 (83.3)	69 (88.5)	0.357
BMI (kg∙m^−2^)	23.6 ± 3.2	23.7 ± 3.2	23.3 ± 3.4	0.251	23.2 ± 3.4	23.2 ± 3.5	0.926
ASA ≥ III	209 (12.6)	206 (13.1)	3 (3.4)	0.008	2 (2.6)	2 (2.6)	1.000
Hypertension	416 (25.1)	338 (24.7)	28 (31.8)	0.134	12 (15.4)	22 (28.2)	0.052
Diabetes mellitus	117 (7.1)	105 (6.7)	12 (13.6)	0.013	6 (7.7)	6 (7.7)	1.000
Coronary artery disease	75 (4.5)	69 (4.4)	6 (6.8)	0.286	2 (2.6)	2 (2.6)	1.000
Alcohol use	972 (58.6)	917 (58.4)	55 (62.5)	0.444	43 (55.1)	46 (59.0)	0.628
Smoking history	1071 (64.6)	1009 (64.2)	62 (70.5)	0.235	47 (60.3)	55 (70.5)	0.178
Preoperative chemoradiotherapy	130 (7.8)	128 (8.1)	2 (2.3)	0.046	3 (3.8)	2 (2.6)	0.649
Preoperative Laboratory							
GLU (mmol/L)	5.5 ± 1.0	5.5 ± 1.0	5.4 ± 1.0	0.503	5.3 ± 0.7	5.4 ± 0.9	0.570
Cr (μmol/L)	75.9 ± 14.1	75.8 ± 14.1	77.4 ± 13.6	0.319	76.7 ± 12.5	77.1 ± 13.9	0.832
ALB (g/L)	43.9 ± 3.3	43.9 ± 3.3	43.8 ± 3.3	0.599	43.6 ± 3.4	43.6 ± 3.2	0.930
Hct (%)	42.4 ± 4.5	42.4 ± 4.5	42.6 ± 4.0	0.766	42.6 ± 3.5	42.6 ± 4.0	0.990
Total fluids (mL)	2657 ± 761	2649 ± 761	2809 ± 747	0.054	2710 ± 698	2736 ± 692	0.818
Total crystalloid (mL)	1896 ± 615	1881 ± 613	2160 ± 593	<0.001	2071 ± 561	2100 ± 529	0.736
Total colloid (mL)	668 ± 375	672 ± 380	585 ± 265	0.004	603 ± 305	577 ± 268	0.578
Operation time (min)	249 ± 95	248 ± 95	255 ± 90	0.537	262 ± 88	245 ± 80	0.212
Intraoperative hypotension ^a^	275 (16.6)	266 (16.9)	9 (10.2)	0.100	8 (10.3)	9 (11.5)	0.797
Intraoperative hypoxemia ^b^	227 (13.7)	214 (13.6)	13 (14.8)	0.760	11 (14.1)	11 (14.1)	1.000
Blood transfusion	225 (13.6)	216 (13.7)	9 (10.2)	0.348	5 (6.4)	7 (9.0)	0.548
Tumor location				0.002			0.876
Upper third	161 (9.7)	155 (9.9)	6 (6.8)		3 (3.8)	5 (6.4)	
Middle third	729 (43.9)	686 (43.7)	43 (48.9)		39 (50.0)	38 (48.7)	
Lower third	449 (27.1)	415 (26.4)	34 (38.6)		32 (41.0)	30 (38.5)	
Gastroesophageal junction	320 (19.3)	315 (20.1)	5 (5.7)		4 (5.1)	5 (6.4)	
Tumor histology				0.116			0.585
Adenocarcinoma	378 (22.8)	365 (23.2)	13 (14.8)		12 (15.4)	13 (16.7)	
Squamous	1177 (70.9)	1106 (70.4)	71 (80.7)		60 (76.9)	62 (79.5)	
Other	104 (6.3)	100 (6.4)	4 (4.5)		6 (7.7)	3 (3.8)	
Tumor stage				0.085			0.346
I	302 (18.2)	292 (18.6)	10 (11.4)		10 (12.8)	8 (10.3)	
II	486 (29.3)	450 (28.6)	36 (40.9)		27 (34.6)	31 (39.7)	
III	714 (43.0)	677 (43.1)	37 (42.0)		31 (39.7)	35 (44.5)	
IV	5 (0.3)	5 (0.3)	0 (0)		0 (0)	0 (0)	
NA	152 (9.2)	147 (9.4)	5 (5.7)		10 (12.8)	4 (5.1)	
Minimally invasive approach	321 (19.3)	306 (19.5)	15 (17.0)	0.574	14 (17.9)	13 (16.7)	0.832
Surgical procedure				0.902			0.561
Sweet	1136 (68.5)	1077 (68.6)	59 (67.0)		50 (64.1)	55 (70.5)	
Ivor-Lewis	148 (8.9)	139 (8.8)	9 (10.2)		11 (14.1)	7 (9.0)	
Three-incision	375 (22.6)	355 (22.6)	20 (22.7)		17 (21.8)	16 (20.5)	
Surgeon experience				<0.001			0.872
High-volume ^c^	1099 (66.2)	1058 (67.3)	41 (46.6)		35 (44.9)	36 (46.2)	
Low-volume ^d^	560 (33.8)	513 (32.7)	47 (53.4)		43 (55.1)	42 (53.8)	

Values are expressed as mean ± standard deviation or number of patients (n, %)

*Abbreviations*: *S* sevoflurane, *P* propofol, *BMI* body mass index, *ASA* American Society of Anesthesiologist, *GLU* glucose, *Cr* serum creatinine, *ALB* albumin, *Hct* hematocrit, *NA* not available

^a^Intraoperative hypotension: lowest systolic blood pressure < 80 mmHg or use of any vasopressor during operation

^b^Intraoperative hypoxemia: arterial oxygen saturation by pulse oximetry <90%

^c^High-volume: ≥100 esophagectomy in three years

^d^Low-volume: <100 esophagectomy in three years

Data were reported as means ± standard deviation (SD) or frequency and percentage (n, %). The Student t test, Mann-Whitney U test, Chi-square test, or Fisher’s exact test was performed for statistical analysis when appropriate. Propensity score matching analysis was also used to allow an unbiased comparison. Patients in propofol group were matched with those in sevoflurane group at a ratio of 1:1 using the nearest neighbor matching with a caliper of 0.001. Besides, we also conducted univariate and multivariate logistic regression analysis to identify independent risk factors for PP. Variables with a *P* < 0.1 in univariate analysis were entered in multivariate logistic regression model. Odd ratios (OR) with 95% confidence intervals (CI) were calculated. A value of *P* < 0.05 was considered statistically significant. All statistical analysis were conducted using the SPSS software (Statistical Package for the Social Sciences, version 22.0, SPSS Inc., Chicago, IL).

## Results

### Study population

In the study period, we identified a total of 2321 patients through our electronic medical records. Of these patients, 662 were excluded from this study according to our exclusion criteria, including 36 patients with missing data and 626 patients anesthetized with anesthetics other than sevoflurane or propofol. Thus, 1659 patients were included in the final analysis. The mean age was 60.7 ± 8.4 years old with 83.4% (*n* = 1383) being male. Among the included 1659 patients, 1571 (94.7%) received sevoflurane inhalational anesthesia while only 88 (5.3%) received propofol intravenous anesthesia. Overall, 158 (9.5%) patients developed postoperative pneumonia. In our cohort of 1659 patients, 92 (5.5%) were admitted or readmitted to the ICU. Only 38 (2.3%) patients did not meet the extubation standards, 33 (2.0%) were re-intubated for mechanical ventilation and 2 (0.1%) received noninvasive ventilation.

### Anesthetic type

The baseline characteristics and intraoperative parameters of the included patients according to the anesthetic type were presented in Table 1. Before propensity matching, there was an uneven distribution of confounders between sevoflurane and propofol groups. More patients experienced preoperative chemoradiotherapy (*P* = 0.046) and less had a history of diabetes mellitus (*P* = 0.013) in sevoflurane group compared with propofol group. Besides, patients in the propofol group were infused more crystalloid (*P* < 0.001) and less colloid (*P* = 0.004) than those in sevoflurane group. There were also significant differences in ASA classification (*P* = 0.008), tumor location (*P* = 0.002) and surgeon experience (*P* < 0.001) between two groups. To allow an unbiased comparison, a propensity score matching analysis was conducted to minimize intergroup differences in some cofounders [20]. After propensity matching, 78 patients in propofol group were successfully matched with 78 patients in sevoflurane group with all *P* values >0.05 (Table 1), ensuring an even distribution of confounders between two groups.

Table 2 showed comparisons of postoperative clinical outcomes before and after propensity score matching. Before propensity score matching, 151 (9.6%) patients in sevoflurane group and 7(8.0%) in propofol group developed PP. After propensity score matching, the incidences of PP were 7.7 and 6.4% in sevoflurane group and propofol group, respectively. No statistically significant difference in the incidence of PP was found between sevoflurane and propofol groups either before (*P* = 0.606) or after propensity score matching (*P* = 0.754). Although the average postoperative hospital stay was significantly longer in sevoflurane group compared with propofol group before propensity score matching (*P* = 0.003), it was not significantly different between two groups after propensity score matching (*P* = 0.307). The other postoperative clinical outcomes also showed no difference between two groups either before or after propensity score matching.

**Table 2:** Comparison of postoperative clinical outcomes before and after propensity score matching between sevoflurane group and propofol group

Postoperative Outcomes	Before Matching			After Matching		
	Group S (n = 1571)	Group P (n = 88)	*P*-value	Group S (n = 78)	Group P (n = 78)	*P*-value
Postoperative pneumonia	151 (9.6)	7 (8.0)	0.606	6 (7.7)	5 (6.4)	0.754
ICU admission	88 (5.6)	4 (4.5)	0.674	4 (5.1)	2 (2.6)	0.405
Reintubation	33 (2.1)	0 (0)	0.170	2 (2.6)	0 (0)	0.155
Reoperation	34 (2.2)	0 (0)	0.163	1 (1.3)	0 (0)	0.316
Total hospital stay ^a^	20 ± 12	19 ± 7	0.061	19 ± 7	18 ± 6	0.566
Postoperative hospital stay ^b^	15 ± 11	13 ± 6	0.003	13 ± 5	12 ± 5	0.307
In-hospital mortality	3 (0.2)	0 (0)	0.682	0 (0)	0 (0)	–

Values are expressed as mean ± standard deviation or number of patients (n, %)

*Abbreviations*: *S* sevoflurane, *P* propofol, *ICU* intensive care unit

^a^Total hospital length of stay: period between the admission and discharge day

^b^Postoperative length of stay: period between the day of surgery and the day of discharge

### Risk factors for PP

The results of both the univariate and multivariate analysis of the risk factors for PP were summarized in Table 3. Variables with *P* value <0.1 in univariate analysis and our interested factor (anesthetic) were entered in multivariate logistic regression model to identify independent risk factors for PP. On multivariate analysis, the independent risk factors for PP were alcohol use (OR 1.513; 95% CI 1.062–2.156), surgical procedure (Sweet: referent; Ivor-Lewis: OR 1.993; 95% CI 1.190–3.337; Three-incision: OR 1.878; 95% CI 1.296–2.722) and surgeon experience (high-volume: referent; low-volume: OR 1.525; 95% CI 1.090–2.135). It was noteworthy that increased body mass index (BMI) appeared to be associated with decreased likelihood of PP (OR 0.935; 95% CI 0.887–0.985). However, neither univariate nor multivariate analysis showed that propofol was associated with the risk of developing PP.

**Table 3:** Univariate and Multivariate analyses of factors associated with postoperative pneumonia

Characteristics	Univariate			Multivariate		
	OR	95% CI	*P*-value	OR	95% CI	*P*-value
BMI	0.933	0.886–0.983	0.010	0.935	0.887–0.985	0.012
Alcohol use	1.592	1.122–2.261	0.009	1.513	1.062–2.156	0.022
Total fluids	1.000	1.000–1.000	0.071			
Total crystalloid	1.000	1.000–1.000	0.096			
Blood transfusion	1.570	1.026–2.401	0.038			
Operation time	1.003	1.001–1.004	0.001			
Anesthetic			0.607			
Sevoflurane	1	Referent				
Propofol	0.813	0.369–1.791				
Tumor location			0.080			
Gastroesophageal junction	1	Referent				
Upper third	2.128	1.109–4.081				
Middle third	1.797	1.079–2.993				
Lower third	1.467	0.840–2.561				
Tumor histology			0.090			
Adenocarcinoma	1	Referent				
Squamous	1.594	1.027–2.475				
Other	1.128	0.495–2.572				
Minimally invasive approach	1.417	0.965–2.080	0.076			
Surgical procedure			<0.001			0.001
Sweet	1	Referent		1	Referent	
Ivor-Lewis	2.019	1.211–3.367		1.993	1.190–3.337	
Three-incision	1.922	1.330–2.777		1.878	1.296–2.722	
Surgeon experience			0.010			0.039
High-volume ^a^	1	Referent		1	Referent	
Low-volume ^b^	1.550	1.111-2.161		1.525	1.090–2.135	

*Abbreviation*: *BMI* body mass index, *OR* odd ratios, *CI* confidence intervals

^a^High-volume: ≥100 esophagectomy in three years

^b^Low-volume: <100 esophagectomy in three years

## Discussion

We conducted this retrospective single-center study to explore the relationship between anesthetic agent and postoperative pneumonia in patients who underwent elective esophageal cancer surgery. Overall, the incidence of PP was 9.6% in sevoflurane group compared with 8.0% in propofol group. Neither the entire cohort nor the propensity score-matched cohort showed any statistically significant difference in PP incidence between two groups.

Propofol and sevoflurane are common general anesthetics used in clinical practice. Some clinical researches have investigated the pulmonary inflammatory-modulating effects of sevoflurane and propofol during OLV. Their results suggested that sevoflurane suppressed the local alveolar inflammatory responses and reduced inflammatory mediators release in patients undergoing OLV, resulting in better clinical outcomes in thoracic surgery [13, 21, 22]. A recent meta-analysis by Sun et al. also showed that inhalational anesthetics might be preferable for OLV during thoracic anesthesia due to their protective effects of attenuating inflammatory responses when compared with propofol-based intravenous anesthesia [23].

However, previous studies mainly addressed the effects of anesthetics on release of inflammatory markers without showing enough data on postoperative clinical outcomes. In addition, esophagectomy might produce greater systemic stress response and less alveolar injury than pulmonary surgery. To our knowledge, few studies have systematically compared the effects of propofol and sevoflurane on the development of PP after esophageal surgery. In our study, we found that two different anesthesia methods had similar effects on the PP rate in patients undergoing esophagectomy. This finding was in accordance with that of Lee’s study, where sevoflurane was not proven to be superior to propofol in lowering postoperative pulmonary complications although the former attenuated increases in inflammatory markers at the end of esophageal operations [9]. It seems that inflammatory-modulating effects of sevoflurane and propofol may not be persistent in the postoperative period [9, 24].

In this study, we also demonstrated that alcohol use, surgical procedure and surgeon experience were independent risk factors of PP while patients with increased BMI appeared to be associated with less likelihood of PP. As BMI of most patients in this study remained within normal range, the effect of BMI on PP should be considered as merely statistical significance, the corresponding clinical relevance may require further investigation. COPD and preoperative pulmonary infections are determining factors in PP development. However, these diseases were rare in our hospital. Only 14 (0.8%) patients (14 in sevoflurane group and 0 in propofol group, *P* = 0.374) were diagnosed with COPD according to their medical history and pulmonary function and no patients had pulmonary infections prior to operation. As for patients with preoperative pulmonary infections, they were transferred to general hospitals for treatment till the symptoms and signs were completely improved. Otherwise, they would not actually be admitted for surgery. Our findings as well as previous published results have also indicated that patients’ physical status and operation correlative factors affected the development of PP more than anesthetics did after esophagectomy.

Several limitations should be considered when interpreting the results in our study. First, certain confounding factors may affect the results given the retrospective design of our study despite the application of propensity score matching analysis. Secondly, we only recruited the patients with confirmed diagnosis of postoperative pneumonia which was indicated explicitly in our electronic medical records. Thirdly, COPD and preoperative pulmonary infections were not considered as comorbidities in this study as these diseases were rare in our hospital. As a specialized cancer center, most patients in our hospital were in better conditions and of less comorbidity than those in general hospitals. Besides, we were lack of data about those with preoperative pulmonary infections who returned to our hospital for surgery after effective treatment. Fourthly, we did not take into account the quality and the type of analgesia (e.g. different opioids or analgesic schemes) because all patients in our study were given intercostal nerve block and patient-controlled intravenous analgesia, and the surgeons would administrate additional analgesics when patients complained of pain. Moreover, postoperative physical therapy such as chest percussion, sputum suctioning, etc., seemed to interfere with pain assessment. Fifthly, patients’ blood samples were not regularly obtained to measure levels of inflammatory markers such as IL-6, IL-8 and IL-10. Although we hypothesized that the anesthetics may alter the postoperative inflammation, there were no data available to suggest postoperative inflammation alteration given the retrospective and observational design of our study. We only compared the effect of anesthetics on the incidence of postoperative pneumonia following esophagectomy. Sixthly, postoperative residual curarization (PORC) was a relevant factor for the development of PP [25]. Although all the extubated patients stayed in PACU for at least 30 min and were discharged from PACU after reaching the discharge criteria, there may be still a risk of morbidity in PORC after tracheal extubation under the guidance of only clinical judgment. However, train-of-four (TOF) stimulation monitor was not regularly applied in our hospital and we were lack of relevant data.

## Conclusions

Postoperative pneumonia occurred in 9.5% of patients after esophagectomy in our hospital. Independent predictors of postoperative pneumonia include alcohol use, surgical procedure and surgeon experience. Anesthetic type does not influence the PP risk after esophageal surgery for cancer.

## Abbreviations

ALB: Albumin
ASA: American Society of Anesthesiologist
BMI: Body mass index
CI: Confidence intervals
COPD: Chronic obstructive pulmonary disease
Cr: Serum creatinine
GLU: Glucose
Hct: Hematocrit
ICU: Intensive care unit
NA: Not available
OLV: One-lung ventilation
OR: Odd ratios
P: Propofol
PACU: Postanesthesia care unit
PEEP: Positive end expiratory pressure
PORC: Postoperative residual curarization
PP: Postoperative pneumonia
S: Sevoflurane
SD: Standard deviation
TOF: train-of-four
